# Early course of microcirculatory perfusion in the eye and digestive tract during experimental sepsis

**DOI:** 10.1186/cc10810

**Published:** 2012-03-20

**Authors:** A Pranskunas, R Rasimaviciute, E Milieskaite, A Vitkauskiene, P Dobozinskas, V Veikutis, Z Dambrauskas, D Vaitkaitis, V Pilvinis

**Affiliations:** 1 Lithuanian University of Health Sciences, Kaunas, Lithuania; 2 Institute of Cardiology, Lithuanian University of Health Sciences, Kaunas, Lithuania

## Introduction

Studies show that sublingual mucosa is a reproducible part for small intestine mucosal microcirculatory perfusion in sepsis, when they are not exposed by local factors. However, it is of great interest how sublingual microcirculation can reflect other beds of microcirculation. The aim of the study is to evaluate and compare the microcirculatory perfusion of potentially available parts of the body, such as sublingual mucosa, conjunctiva of the eye, mucosa of jejunum and rectum, at the same time points during experimental sepsis.

## Methods

Pigs were randomly assigned to sepsis (*n *= 9) and sham (*n *= 4) groups. The sepsis group received a fixed dose of live *Escherichia coli *infusion over 1 hour. Animals were observed 5 hours after the start of *E. coli *infusion. In addition to systemic hemodynamic assessment, we performed conjunctival, sublingual, jejunal and rectal evaluation of microcirculation using sidestream dark-field videomicroscopy at the same time points: at baseline, 3 and 5 hours after the start of live *E. coli *infusion. Assessment of microcirculatory parameters of convective oxygen transport (microvascular flow index (MFI), proportion of perfused vessels (PPV)) and diffusion distance (perfused vessel density, total vessel density) was done using a semiquantitative method.

## Results

Infusion of *E. coli *resulted in a hypodynamic state of sepsis despite fluid administration. Significant decreases in MFI and PPV of small vessels were in sublingual, conjunctival, jejunal and rectal lodges 3 and 5 hours after the start of *E. coli *infusion in comparison to baseline variables. Correlation between sublingual and conjunctival (*r *= 0.80, *P *= 0.036), sublingual and jejunal (*r *= 0.94, *P *= 0.005), sublingual and rectal (*r *= 0.79, *P *= 0.03) MFI was observed 3 hours after onset of sepsis. There was no correlation in change of MFI and PPV between sublingual mucosa and other evaluated regions. However, the sublingual mucosa exhibited the most pronounced alterations of microcirculatory flow in comparison to conjunctival, jejunal and rectal mucosa microvasculature (*P *< 0.05).

## Conclusion

Microcirculatory alterations were observed in all investi-gated lodges, including sublingual, jejunal and rectal mucosa, and conjunctiva of the eye at the same time point during experimental sepsis. There is a clear association between sublingual microcirculation and conjunctival, jejunal or rectal microcirculation in the very early course of an extreme hypodynamic state of sepsis.

